# Establishing Detection Methods for Okadaic Acid Aptamer–Target Interactions: Insights from Computational and Experimental Approaches

**DOI:** 10.3390/foods14050854

**Published:** 2025-03-02

**Authors:** Wenchong Shan, Jiefang Sun, Runqing Liu, Jing Wang, Bing Shao

**Affiliations:** 1 National Nanfan Research Institute (Sanya), Chinese Academy of Agricultural Sciences, Sanya 572024, China; shanwenchong@163.com; 2 Key Laboratory of Agro-Product Quality and Safety, Institute of Quality Standard and Testing Technology for Agro-Products, Chinese Academy of Agricultural Sciences, Beijing 100081, China; 3 Key Laboratory of Agri-Food Safety and Quality, Ministry of Agriculture of China, Beijing 100081, China; 4 Beijing Key Laboratory of Diagnostic and Traceability Technologies for Food Poisoning, Beijing Center for Disease Prevention and Control, Beijing 100013, China; sunjf2001@163.com (J.S.); lrq_srunh@163.com (R.L.); 5 National Key Laboratory of Veterinary Public Health Security, Beijing Key Laboratory of Detection Technology for Animal-Derived Food Safety, and Beijing Laboratory for Food Quality and Safety, College of Veterinary Medicine, China Agricultural University, Beijing 100193, China; 6 Food Laboratory of Zhongyuan, Luohe 462300, China

**Keywords:** okadaic acid, aptamer, binding interactions, food safety, molecular docking simulations, marine toxins

## Abstract

The binding interactions between okadaic acid (OA) aptamers and OA molecules are crucial for developing effective detection methods. This study aims to identify the recognition site and establish a reliable detection protocol through computational simulations and experimental validations. After determining the target sequence (OA-2), molecular docking simulations using Sybyl-X and H-dock were conducted to predict the binding affinity and interaction sites of OA aptamers with their targets. These predictions were subsequently validated through experiments based on the Förster resonance energy transfer (FRET) principle. The combined approach not only confirmed the computational predictions, identifying the “major region” as the recognition basis of OA-2, but also provided deeper insights into the binding mechanisms. Subsequently, a classical AuNPs-aptamer colorimetric detection method was established based on the OA-2 sequence and applied to the detection of real shellfish samples, achieving a limit of quantification (LOQ) of 5.0 μg kg^−1^. The recoveries of OA in spiked samples ranged from 79.0% to 122.9%, with a relative standard deviation (RSD) of less than 14.7%. The results of this study contribute to the development of robust detection methods for OA aptamer–target interactions, enhancing the potential for practical applications in toxin detection and monitoring.

## 1. Introduction

An aptamer, derived through the systematic evolution of ligands by exponential enrichment (SELEX) [1], is a short single-stranded nucleic acid sequence capable of binding to specific ligands due to its three-dimensional structure [2]. Through the screening of large oligonucleotide libraries, aptamers have been successfully selected against a wide range of analytes, including metal ions [3], small molecules [4], proteins [5], toxins [6], miRNAs [7], and even whole cells [8]. Due to their high specificity, aptamers are widely used as recognition elements in biosensors, where their performance is closely linked to their affinity for the target ligand [9].

The affinity of aptamers, often expressed using the dissociation constant (*Kd*) [10,11], is a key metric for evaluating their performance. Methods such as the enzyme-linked immunosorbent assay (ELISA), surface plasmon resonance (SPR), isothermal titration calorimetry (ITC), microscale thermophoresis (MST), and fluorescence spectrometry are commonly used to determine *Kd* values. However, due to differences in recognition conditions and measurement principles among these methods, variations in *Kd* values with an order-of-magnitude divergence can be observed for the same aptamer [12].

Marine toxins, produced by algae, are biotoxins commonly found in shellfish and fish, often coexisting as multiple toxins. Given their potent toxicity, aptamers have emerged as promising recognition probes for detecting these toxins. While sequences of marine toxin aptamers have been reported, studies on their binding sites and recognition mechanisms remain limited. Investigating these aspects can reveal the interaction principles between aptamers and their targets, providing a foundation for designing more efficient and specific aptamers. Systematic research in this area is of considerable scientific and practical importance.

Okadaic acid (OA), a major diarrhetic shellfish toxin, poses a significant public health risk, making its detection critical for food safety. Advancing OA detection methods is crucial for enhancing food industry practices and safeguarding consumer health. To better understand the recognition mechanisms and binding regions of aptamers, molecular docking simulations have become widely employed [13,14]. These simulations rely on three-dimensional structures of aptamers and ligands, but variations in software tools and parameters can lead to differing results. In this study, we used SYBYL-X [15] and H-dock [16] to simulate aptamer–OA interactions. The results were analyzed and validated through Förster resonance energy transfer (FRET) experiments, confirming the aptamer’s binding site.

Finally, a colorimetric detection biosensor was developed by combining gold nanoparticles (AuNPs) with the selected aptamer [17]. The aptamer–ligand interactions modulate the salt tolerance of AuNPs, causing a visible color change in the solution. By analyzing absorbance spectra, the local concentration of OA was accurately quantified. This approach enables the rapid detection of OA, particularly in food safety applications. Overall, this study advances our understanding of aptamer–target interactions and contributes to the development of practical toxin detection methods.

## 2. Materials and Methods

### 2.1. Materials and Apparatus

All DNA sequences were synthesized by Suzhou Genewiz Biotechnology Co., Ltd. (Suzhou, China), in dry powder form and purified by high-performance liquid chromatography (HPLC) with a purity of 95%. The detailed DNA sequences are provided in Table S1. Each aptamer was dissolved in buffer solutions as specified in the original literature for the corresponding primer and stored at −20 °C, with minimal freeze–thaw cycles to maintain stability and prevent degradation. Saxitoxin (STX), decarbamoylsaxitoxin (dcSTX), neosaxitoxin (NEO-STX), microcystin YR (MC-YR), okadaic acid (OA), dinophysistoxin-1 (DTX-1), brevetoxin-2 (BTX-2), and brevetoxin-3 (BTX-3) were procured from the National Research Council of Canada (NRCC, Halifax NS, Canada). Tetrodotoxin (TTX), microcystin LR (MC-LR), microcystin LA (MC-LA), microcystin RR (MC-RR), gonyautoxin-1/4 (GTX-1/4), and domoic acid (DA) were purchased from Pribolab Co., Ltd. (Qingdao, China). Trisodium citrate dihydrate (99.5%), NaCl (99.5%), and other inorganic salts used in buffers were obtained from Sinopharm Chemical Reagent Co., Ltd. (Shanghai, China). DEPC-treated water and HAuCl_4_ (99.99%) were purchased from Sigma-Aldrich (St. Louis, MO, USA). The buffers used in this work are listed in Table S2. Water was purified with a Milli-Q purification system (18.2 MΩ, DI water, Milli-Q) and buffers were made fresh each week.

Fluorescence intensity was measured using an EnVision^®^ 2105 multimode plate reader (PerkinElmer, Inc., Waltham, MA, USA). MST experiments were performed using a Monolith NT.115 (NanoTemper, Munich, Germany) with Mo NT.115 capillaries. The shellfish tissue sample was homogenized using a FastPrep 24 sample preparation system (MP Biomedical, Santa Ana, CA, USA). All statistical analyses were performed using Origin 2018 software (Origin Lab, Northampton, MA, USA).

### 2.2. FRET-Aptamer Assay

All the reported marine toxin aptamers were summarized and synthesized, and were labeled with FAM at the 5′-end and BHQ-1 at the 3′-end to construct the FRET-aptamer. Each sequence was diluted with its respective buffer and annealed with incubation at 95 °C in a water bath for 10 min followed by an ice bath for 10 min. The analyte stock solution for each aptamer was diluted to a series of concentrations of working solution (i.e., 0.5, 1.0, 2.0, 4.0, 5.0, 6.0, 8.0, and 10.0 μmol L^−1^). Then, 90 μL FRET-aptamer was mixed with 10 μL of different concentrations of targets, and the solution was transferred to a 37 °C incubator and incubated for 60 min in the dark. The fluorescence intensity (FI) was measured, and curve fitting, as well as data analysis, were performed to calculate the *Kd* values of each sequence.

### 2.3. Microscale Thermophoresis Assay

The aptamers were labeled with Cy5-dye at the 5′ end and kept a constant concentration of 0.1 nmol L^−1^ in all MST experiments. The procedure of MST was adapted from a previous study [18] and can be briefly summarized as follows. With the help of MO. Affinity Analysis v2.3, the concentration of Cy5-aptamer was optimized to make its FI value between 5000 and 20,000, and this concentration was used to bind with the target. The target concentration was varied by 1:1 gradient dilution 16 times. Then, the aptamer and the corresponding target solution were incubated with a mixing ratio of 1:1 for 5 min at room temperature and loaded into standard treated capillaries. The software MO. Affinity Analysis v2.3 was used for data analysis, and the curves were fitted by the *Kd* algorithm.

### 2.4. Docking Simulation by SYBYL-X

The secondary and tertiary structures (PDB format) of aptamers were built using the Mc-fold/Mc-sym pipeline (https://major.iric.ca/MC-Pipeline/, accessed on 8 January 2025) [19]. The python tool was utilized for automated download and renamed for the tertiary structures (see the Supplementary Materials, File S1). The 3D structure (mol2 format) of the target molecule was built and minimized by MM2 using Chem 3D software 20.0 [20]. Subsequently, both aptamer and ligand tertiary structures were imported into SYBYL-X for visualization and simulation. Finally, the potential binding sites of the aptamer with ligand-specific recognition were predicted by the molecular docking module surflex-dock in the SYBYL package [21].

### 2.5. Docking Simulation by H-Dock

The secondary structures (*ct* file format) of aptamers were predicted by the UNAFold web server (http://www.unafold.org/, accessed on 8 January 2025) [22]. Then, they were imported into RNA Composer (http://rnacomposer.cs.put.poznan.pl/, accessed on 8 January 2025) [23] to generate the 3D structure (PDB format), and all the T bases were modified to U bases during this process. The target molecule was built using the same method as for the 3D structure. Discovery Studio 2021 [24] was used for visualization and converting U bases to T bases. The docking between aptamers and ligands was performed by using the H-dock server (http://hdock.phys.hust.edu.cn/, accessed on 8 January 2025) [25], which uses an FTT-based docking algorithm.

### 2.6. Detection of OA

Gold nanoparticles (AuNPs) were synthesized using the classical Plech Turkevich method with slight modifications [26]. Briefly, 0.02 g of HAuCl_4_ powder was added to 200 mL of pure water and allowed to boil on a hot plate with magnetic stirring in the dark. Subsequently, 0.06 g of trisodium citrate dihydrate was accurately weighed and dissolved into 6 mL water, which was quickly injected into the boiling solution with continuous stirring and boiling for 15 min. The prepared AuNP solution was condensed by centrifugation at 10,000 rpm for 20 min, and one-fourth of the volume of the lower layer of the solution was retained, which was sonicated for 2 min and stored at 4 °C while protected from light.

The following is a brief overview of the OA detection process. First, 10 μL of OA standard solution and an equal volume of 1 μmol L^−1^ aptamer sequence were mixed and incubated at room temperature for 5 min. Subsequently, the mixed solution and 150 μL of the AuNP solution were incubated together for another 5 min in the dark. After 50 μL of 100 mmol L^−1^ NaCl was added and vortexed for 30 s, the mixture was transferred to a 96-well transparent microplate, and the absorbance at 520 nm and 650 nm was measured.

### 2.7. Real Sample Preparation

Fresh shellfish was bought from the local market, and the tissue was removed from the shell. A DI water rinse was performed on the shellfish tissue mass, and the excess water was removed by touching it with filter paper. An adaptation of the shellfish pretreatment procedure was established from a previous method, which can be summarized as follows. A 2.0 g sample was accurately weighed and homogenized for 30 s. Subsequently, 20 mL of methanol was added and sonicated for 5 min. Then, 1.0 mL of the extraction solution was transferred to a new centrifuge tube and supplemented with 125 μL of 2.5 mol L^−1^ NaOH, and the solution was heated at 76 °C for 40 min, after which we added 125 μL of 2.5 mol L^−1^ HCl to the mixture. Finally, the extraction solution was well mixed and filtered with a 0.22 μm filter for further experimentation.

## 3. Results

### 3.1. Selection of Sequences and Affinity Determination

**Figure 1:**
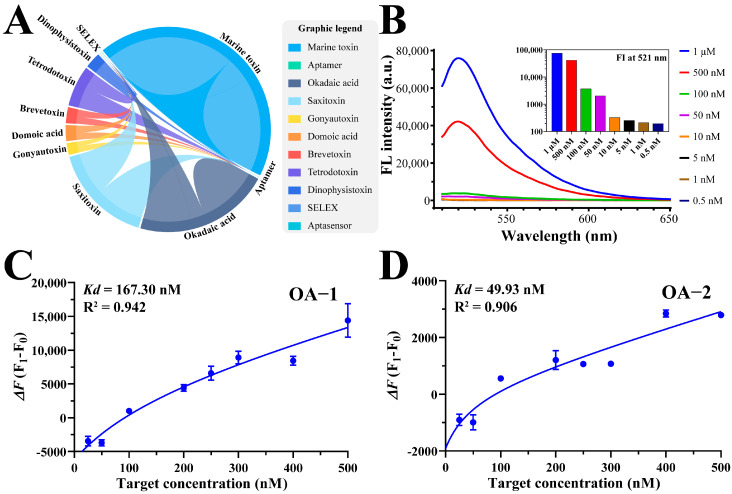
A literature overview of the marine toxin aptamers and fluorescence characteristics of the selected aptamers. (**A**) The publications (through 2022) featured the theme of marine toxin aptamers and were obtained from Web of Science (WoS). (**B**) The fluorescence intensity corresponded to different FAM-OA-1 sequence concentrations. The inset in (**B**) shows the fluorescence intensity at 521 nm for various concentrations, with the *Y*-axis presented on a logarithmic scale (base 10). (**C**,**D**) *Kd* estimation for aptamers binding with individual targets based on FRET measurements for OA-1 and OA-2, respectively.

To ensure the reliability of aptamer sequences for model development, we conducted a comprehensive literature review, identifying 109 studies related to “marine toxin” and “aptamer”. After filtering duplicates, 19 aptamer sequences were selected based on their reported binding affinity and structural features, providing a solid foundation for experimental validation (Figure 1A).

Secondary structure analysis revealed stable stem-loop regions in the selected aptamers (Figure S1), indicating potential structural integrity under experimental conditions. Initial fluorescence quenching optimization using FRET-OA-1 suggested 500 nmol L^−1^ as the optimal aptamer concentration for binding studies (Figure 1B). Both OA-1 and OA-2 aptamers showed strong affinity for okadaic acid, with *Kd* values below 800 nmol L^−1^ (Figure 1C,D).

**Figure 2:**
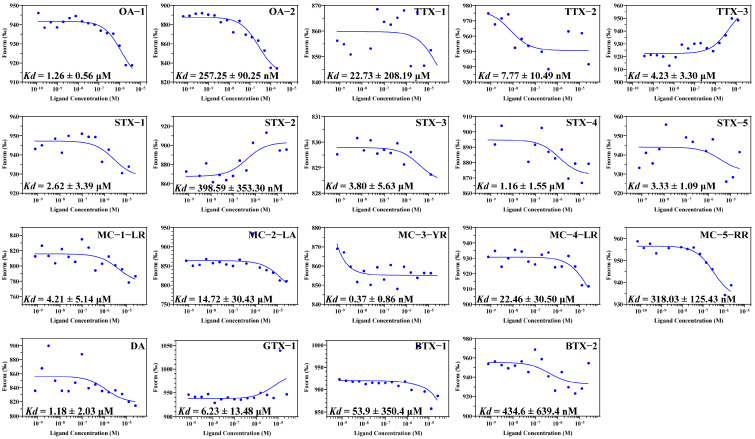
MST measurement results of the 19 toxin aptamer sequences, showing the binding curves of each toxin-aptamer interaction. The upper right corner lists the corresponding aptamer IDs for each marine toxin, while the lower left corner presents the dissociation constants (*Kd*) for the interactions.

While *Kd* values were obtained using FRET, methodological differences, such as fluorophore positioning and aptamer concentration optimization, may result in more variations compared to prior studies. Microscale thermophoresis (MST) provided complementary validation (Figure 2), confirming OA-2 as the highest-affinity aptamer (*Kd* = 257.25 ± 90.25 nmol L^−1^). This sequence was selected for subsequent molecular docking and colorimetric assay development. 

### 3.2. Binding Site Prediction and Verification

To achieve a comprehensive understanding of the binding regions of the OA-2 aptamer, we employed two complementary docking simulation tools, SYBYL-X and H-dock. These tools leverage distinct algorithms, allowing for a more robust analysis of aptamer–target interactions. SYBYL focuses on optimizing small, flexible binding regions with its empirical scoring functions, while H-dock employs a grid-based strategy that excels at exploring broader interaction possibilities. By integrating their predictions, we aimed to overcome the limitations of individual methods and achieve a more comprehensive view of aptamer binding modes.

**Figure 3:**
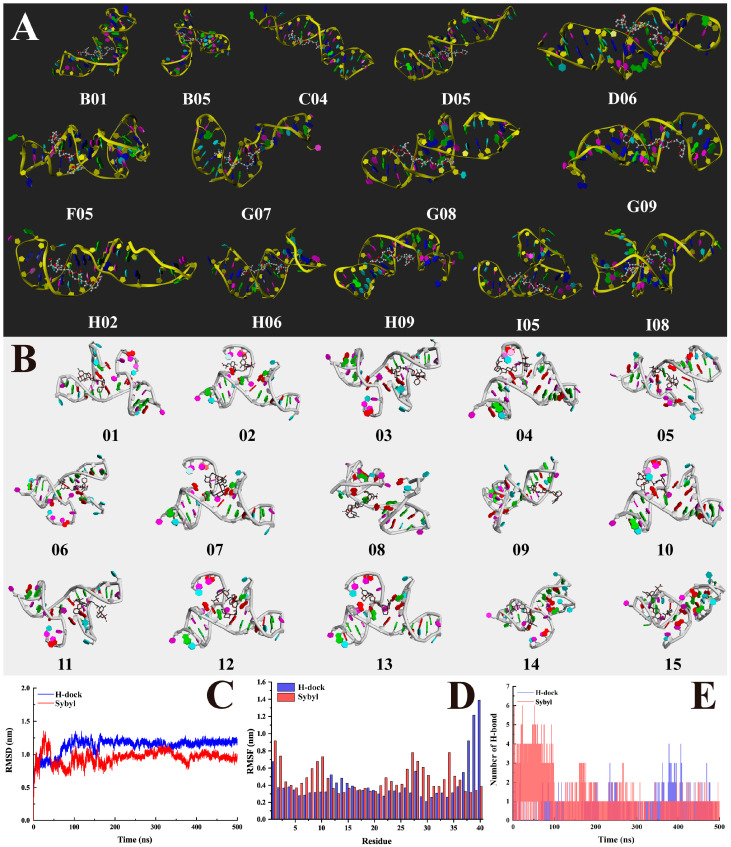
Comparison of the simulated docking results using two different prediction methods. (**A**) The docking simulation modes between OA-2 and OA molecules with CScores ≥ 5.0 according to SYBYL-X. (**B**) The top 15 docking simulation modes between OA-2 and OA molecules according to H-dock. (**C**) The RMSD of the aptamer–target complex over 500 ns for the MD simulations. (**D**) The RMSF of residues in the OA-2 for the MD simulations. (E) The time-resolved hydrogen bonding analysis between the aptamer and target.

SYBYL-X utilizes the scoring model CScore to evaluate ligand–aptamer interactions [27]. Among the initial 100 models generated, 14 docking modes with CScores ≥ 5.0 were selected (Figure 3A). These modes indicated that the OA molecule predominantly binds near the 3′ end of the aptamer, with hydrogen bonds serving as the primary interaction force. A homology analysis of these predicted sites identified a consensus sequence, ‘G*G*CGCTACCACC*’, located in the stem region of the aptamer (Figure S2).

H-dock predicted a broader distribution of binding interactions across the aptamer sequence. Out of the 100 docking models generated, the 15 highest-ranked models revealed strong hydrogen bonding and *Pi*–alkyl interactions between the aptamer and OA (Figure 3C). Homology analysis identified the base ‘6A’ as the most frequently occurring binding site, with a total of 33 instances, whereas bases such as ‘29T’ and ‘31G’ were not involved in any binding models. The predicted binding sites spanned both the stem region and a loop structure, highlighting significant differences from the SYBYL predictions.

The GROMACS 2025.0 was used for Molecular dynamics (MD) simulations, and relevant data for the best docking conformations of SYBYL and H-dock were collected over a 500 ns simulation period. The analysis of the root-mean-square deviation (RMSD) values (Figure 3C) revealed that both the H-dock and SYBYL docking models achieved stable conformations after initial fluctuations, with RMSD values stabilizing around 1.0 nm. However, compared to the SYBYL model, the H-dock model exhibited smaller fluctuations and reached stability more quickly, indicating that the H-dock complex was more stable during the simulation. From the root-mean-square fluctuation (RMSF) data (Figure 3D), it was observed that in the H-dock simulation, most residues exhibited lower fluctuations (0.2–0.6 nm), indicating that the structure of the aptamer–target complex was more rigid and highly stable. Meanwhile, in the SYBYL simulation, fluctuations were more pronounced, particularly at residues 10C, 30C, and 35C, where the RMSF values exceeded 0.8 nm, further indicating lower stability compared to H-dock. The analysis of the number of hydrogen bonds (Figure 3E) showed that the H-dock model maintained a consistently stable number of hydrogen bonds (2 to 4), suggesting that the binding interactions remained stable during the MD simulation. In contrast, the SYBYL model exhibited significant fluctuations in hydrogen bond numbers, indicating less stable binding interactions. Based on these findings, we conclude that the H-dock model is more reliable, with stronger and more stable binding interactions.

**Figure 4:**
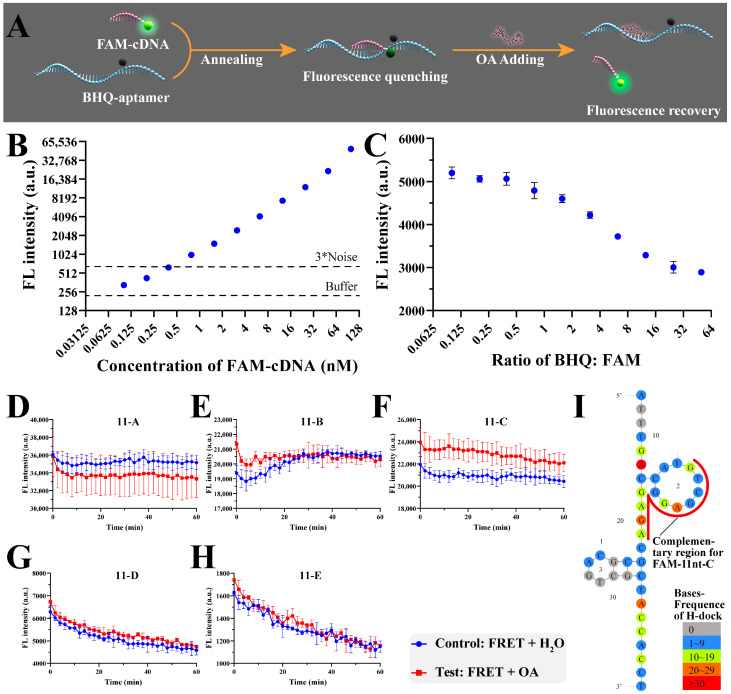
Validation of recognition site localization using the FRET method. (**A**) A schematic diagram of duplexed-FRET complex designing to determine the binding sites of OA-2. (**B**) Concentration optimization of FAM-11nt-n via FI measurement. (**C**) Ratio optimization of FAM-11nt-n and APT-mid-BHQ1 via FI measurement. (**D**–**H**) Fluorescence change in duplexed-FRET complexes in 1 h after OA addition. (**I**) A comparison of the complementary region for FAM-11nt-C and binding sites predicted by H-dock.

To validate these predictions experimentally, complementary DNA (cDNA) sequences were designed based on key predicted regions (Table S3). Initial designs with 15 nt complementary strands showed strong hybridization but failed to exhibit fluorescence recovery upon OA addition, likely due to excessive stability (Figure S3). Subsequent experiments with shorter 11 nt cDNA sequences successfully demonstrated strand dissociation upon OA binding, with the 11-C region showing the most pronounced fluorescence increase (Figure 4). These results confirm the importance of the predicted binding sites, particularly those identified by H-dock.

### 3.3. Establishment of the Classic Colorimetric Method

**Figure 5:**
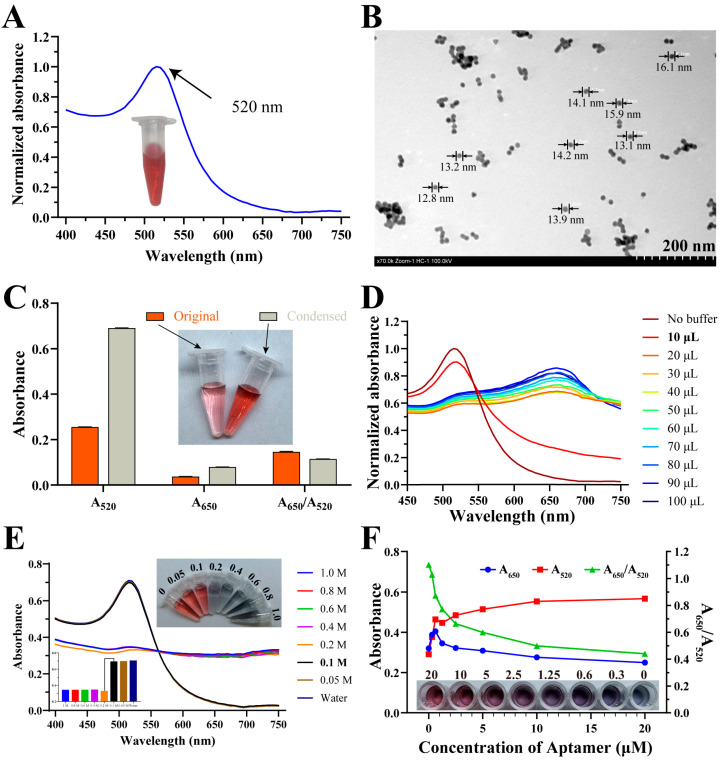
Optimization of aptamer–AuNP colorimetric assay. (**A**) UV-vis absorption spectra of AuNPs. (**B**) TEM characterization of AuNPs. (**C**) Changes in AuNPs before and after centrifugation. (**D**) Volume optimization of BB addition. (**E**) Concentration optimization of NaCl addition with corresponding solution image. Inset graph on left shows fluorescence intensity at 520 nm for various concentrations. (**F**) Concentration optimization of aptamer determined by measuring absorbance with corresponding solution image.

To establish a colorimetric method for OA detection, the OA-2 aptamer was employed due to its short sequence (40 bases) and minimal redundant regions. This label-free strategy relies on the interaction between the aptamer and the target, which influences the adsorption of the aptamer onto the gold nanoparticle (AuNP) surface, thereby affecting the aggregation stability of AuNPs in a salt solution. In the absence of the target, the aptamer stabilizes the AuNPs, maintaining their dispersed state and characteristic purplish-red color, with a maximum absorption wavelength at 520 nm (Figure 5A). However, upon target binding, the aptamer undergoes a conformational change, reducing its affinity for AuNPs and leading to salt-induced aggregation, which results in a colorimetric shift [28].

Dynamic light scattering (DLS) analysis indicated a hydrated particle size of 20.52 ± 0.22 nm, which was consistent with the 12–17 nm particle size observed in the TEM images (Figure 5B). TEM further confirmed that the AuNPs were well dispersed without aggregation. Concentrating the AuNP solution via centrifugation resulted in a deeper red color, with a threefold increase in absorbance at 520 nm and no aggregation-related absorbance increase at 650 nm (Figure 5C).

To optimize the assay conditions, the effects of binding buffer (BB) volume, NaCl concentration, and aptamer concentration were evaluated. Increasing the BB volume resulted in a decrease in A_520_, with 10 μL determined as the optimal volume for maintaining AuNP dispersion (Figure 5D). For NaCl, a concentration of 0.1 mol L^−1^ was identified as the critical threshold, beyond which AuNP aggregation occurred, leading to color changes and the disappearance of the A_520_ peak (Figure 5E). The aptamer concentration significantly affected the AuNPs’ salt tolerance. At higher aptamer concentrations (1–2 μmol L^−1^), the solution color shifted back to purplish-red, accompanied by an increase in A_520_ and a decrease in A_650_ (Figure 5F). Based on these findings, aptamer concentrations of 1 μmol L^−1^ and 2 μmol L^−1^ were tested in subsequent experiments.

Using the optimized conditions, a colorimetric sensor was developed for OA detection. The standard curve constructed with an aptamer concentration of 1 μmol L^−1^ exhibited a broader linear range (1.0–500.0 μg L^−1^) compared to 2 μmol L^−1^ and was selected for further studies (Figure S4). The sensor demonstrated a strong correlation between the log(A_650_/A_520_) ratio and OA concentration (R^2^ = 0.9614), with a detection limit (LOD) of 1.2 μg L^−1^, calculated using the 6S_0_/S formula, where S represents the slope of the linear equation and S_0_ represents the standard deviation of the blank solution.

To further confirm the detection mechanism, TEM imaging was performed to observe AuNP aggregation at different OA concentrations. Without OA, AuNPs remained uniformly dispersed (Figure S5A). Increasing the OA concentrations led to progressive aggregation, with larger clusters forming at higher target levels (Figure S5B). These results confirm the successful construction of the aptamer–AuNP biosensor for OA detection.

### 3.4. Detection of Real Samples

**Figure 6:**
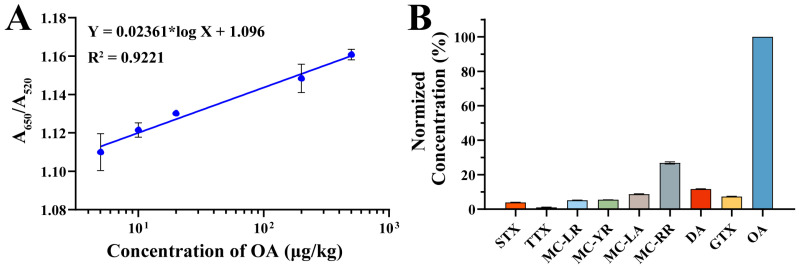
Methodological characterization for aptasensor. (**A**) Relationship between A_650_/A_520_ of proposed aptasensor and OA concentration in shellfish samples. (**B**) Normalized data of this aptasensor with different toxins in same concentration (i.e., STX, TTX, MC-LR, MC-YR, MC-LA, MC-RR, DA, GTX, and OA).

To evaluate the practical application of the aptamer–AuNP biosensor, a series of OA concentrations was added to fresh, negative shellfish samples for testing. The linear equation fitted was *Y* = 0.02361 × log(*X*) + 1.096, where *Y* represents the value of A_650_/A_520_ and *X* the concentration of OA in shellfish samples (Figure 6A). This equation shows a good linear relationship between A_650_/A_520_ and OA in the range of 5.0 μg kg^−1^ to 500.0 μg kg^−1^ (R^2^ = 0.9221), with the limit of quantitation (LOQ) being 5.0 μg kg^−1^. This sensitivity surpasses the reference dose of 160 μg·kg^−1^ established by the European Food Safety Authority (EFSA) [29], indicating the aptasensor’s regulatory compliance.

To assess specificity, 10.0 μg·L^−1^ of OA, STX, MC-LR, MC-YR, MC-LA, MC-RR, DA, and GTX were tested under identical conditions. The standardized results (Figure 6B) showed that other marine toxins did not produce significant signals, demonstrating excellent selectivity for OA detection.

Recovery experiments were conducted by spiking negative shellfish samples with 10.0, 20.0, and 50.0 μg·kg^−1^ of OA. The recoveries ranged from 79.0% to 122.9%, with relative standard deviations (RSDs) below 14.7% (Table 1). This performance aligns with the requirements for food safety testing. Compared to other conventional instrumental methods, the aptasensor offers advantages in terms of simplicity, cost-effectiveness, and ease of use, making it highly suitable for the routine monitoring of OA contamination in shellfish.

## 4. Discussion

It is important to acknowledge that while the aptamer sequences used in this study were synthesized based on previously reported sequences, variations in affinity measurements can arise due to differences in experimental methodologies. In this study, the initial *Kd* values for the 19 aptamers were determined using the FRET method. As a preliminary selection, fluorophore and quencher groups were positioned at the termini of the aptamer sequences. The distance between the fluorophore and the quencher is crucial for FRET efficiency and, consequently, impacts the accuracy of affinity measurements [30]. Variations in their positioning may influence energy transfer efficiency, leading to differences in the observed *Kd* values. Additionally, the optimization of aptamer concentrations in our experiments, particularly the molar ratio between the aptamer and target molecule, may have contributed to slight discrepancies when compared to the original studies. These methodological differences highlight the significance of experimental context when interpreting binding affinity results, underscoring how even minor variations in experimental conditions can lead to notable differences in the measured outcomes. Moreover, while both the FRET and MST methods are widely used to assess aptamer binding affinity, they rely on different principles and experimental setups, which can lead to variations in sensitivity and interpretation. The FRET method depends on the proximity between the fluorophore and the quencher, with fluorescence recovery linked to their spatial arrangement [31]. This can be influenced by the aptamer’s conformational flexibility and binding-induced structural changes. In contrast, MST measures the movement of the aptamer–target complex in solution, with binding affinity inferred from changes in the complex’s thermal mobility [32]. These methodological differences can result in consistent trends in aptamer affinity, but slight discrepancies in the measured *Kd* values.

Although both methods revealed consistent trends in aptamer affinity, the minor differences in *Kd* values underscore the importance of considering experimental context and methodology. Factors such as fluorophore positioning, aptamer concentration, buffer composition, and temperature conditions can all contribute to variations in affinity measurements [33]. These findings suggest that while FRET and MST offer complementary insights into aptamer–target interactions, a comprehensive understanding of binding affinity requires the integration of multiple techniques, each with its inherent strengths and limitations. Future studies should aim to standardize protocols for aptamer labeling, concentration optimization, and experimental conditions to ensure the reproducibility and comparability of results. In addition, incorporating other validation techniques, such as surface plasmon resonance (SPR) or isothermal titration calorimetry (ITC), could further enhance the robustness of the findings and provide additional support for FRET and MST results [34,35]. Computational simulations, including molecular dynamics studies, can also provide insights into the structural dynamics of aptamers and their interactions with target molecules, potentially refining our understanding of their binding mechanisms [36].

The discrepancies in binding site predictions between SYBYL and H-dock reflect their distinct methodologies and scoring principles. The SYBYL focuses on geometric optimization and conformational stability, its scoring functions tend to prioritize structural flexibility, which may not always align with the true nature of the binding interactions [37]. While H-dock has superior ability to evaluate shape complementarity, which is critical for the precise fitting of molecular shapes, especially in nucleic acid-target interactions [16]. The SYBYL’s shape-based similarity algorithm, optimized for small, flexible regions, identified a localized site near the 3’ end. In contrast, H-dock’s grid-based strategy predicted more dispersed binding interactions, accommodating the entire aptamer sequence. These differences emphasize the complementary nature of the two approaches and the necessity of integrating their results for a comprehensive assessment.

Experimental validation further demonstrated that OA-2’s recognition sites are dispersed throughout its sequence, aligning more closely with H-dock predictions. Attempts to truncate the aptamer revealed no improvement in affinity, suggesting that OA-2 represents an already optimized sequence. While SYBYL and H-dock provided valuable insights, the discrepancies highlight the need for more advanced docking tools capable of accommodating flexible nucleic acid structures. These findings lay the groundwork for future optimization strategies and support the robust integration of computational and experimental methods in aptamer research.

In recent years, artificial intelligence (AI)-assisted biosensing has gained significant attention due to its ability to enhance sensor performance, data analysis, and real-time detection through machine learning algorithms [38]. Advanced techniques such as deep learning have been applied to various aspects of aptamer-based sensor development, including aptamer selection, binding site prediction, and sensor platform optimization. AI-driven methods have been successfully integrated into colorimetric biosensors for rapid visual detection and electrochemical sensors for highly sensitive and selective analyte recognition [39,40], demonstrating their potential to significantly improve detection accuracy, minimize false positives, and enable real-time data processing. These advancements highlight the growing synergy between AI and label-free aptasensors, paving the way for more efficient and intelligent biosensing systems. Future research should focus on refining AI algorithms specifically designed for aptamer-based sensors, broadening their applicability to complex sample matrices, and developing portable, user-friendly diagnostic platforms that can facilitate on-site and point-of-care testing.

## 5. Conclusions

This study utilized previously reported marine toxin aptamer sequences to conduct homogeneous fluorescence quenching screening, ultimately identifying OA-2 as the optimal template sequence through MST analysis. Building on this, binding site predictions and computer-simulated docking were performed for OA-2. By designing and testing complementary sequence fragments, the recognition region was precisely determined, providing valuable insights into the recognition mechanism of this aptamer. Furthermore, leveraging the unique structural and functional characteristics of the OA-2 sequence, a classic aptamer–AuNP colorimetric biosensor was developed. This sensor demonstrated excellent performance in detecting OA in real shellfish samples, showcasing its potential for practical applications in food safety monitoring. Despite these promising results, this study also highlights the limitations of current computational docking tools. Factors such as force field selection, ionic strength, and environmental temperature significantly influence the accuracy of predictions, making it challenging to precisely model nucleic acid and small-molecule interactions in complex environments. In the future, precise simulations of molecular docking under specific conditions could greatly enhance the predictive capabilities of aptamer-based biosensor design.

## Figures and Tables

**Table 1:** Recoveries of different concentrations of OA added to shellfish samples.

Concentration Spiked (μg kg^−1^)	Concentration Calculated (μg kg^−1^)	Recoveries (%)	RSD (%)
10.0	12.3 ± 0.15	122.9	1.2
20.0	22.4 ± 0.55	112.1	2.5
50.0	54.8 ± 5.80	79.0	14.7

## Data Availability

The data available in this study are available in Figure 1, Figure 2, Figure 3, Figure 4, Figure 5 and Figure 6; Table 1; and the Supplementary Materials.

## Supplementary Materials

The following supporting information can be downloaded at: https://www.mdpi.com/article/10.3390/foods14050854/s1, Table S1: Details of FRET-aptamer for marine toxins; Table S2: Details of binding buffers used in this study; Table S3: Details of other involved sequences; Figure S1: Secondary structures of marine toxin aptamers with UNAfold prediction with color auto-labeled; Figure S2: Homology analysis of high-score sequences for SYBYL-X core regions prediction used DNAMAN; Figure S3: Predicted secondary structures and fluorescence intensity changes in FRET complexes; Figure S4: Performance and visual responses of aptasensors with varying aptamer and OA concentrations; Figure S5: TEM images of AuNP–aptamer system; File S1: Details of Python code for batch download.

## Abbreviations

The following abbreviations are used in this manuscript:

OA	Okadaic acid
FRET	Förster resonance energy transfer
MST	Microscale thermophoresis
AuNPs	Gold nanoparticles
FI	Fluorescence intensity
cDNA	Complementary deoxyribonucleic acid
BB	Binding buffer
LOQ	Limit of quantification
RSD	Relative standard deviation
