# Supplementary material for: Establishing Detection Methods for Okadaic Acid Aptamer–Target Interactions: Insights from Computational and Experimental Approaches
Source: Foods. 2025 Mar 2;14(5):854. doi: 10.3390/foods14050854 (PMC11898563; doi:10.3390/foods14050854)
Supplement: Supplementary file 1 [file foods-14-00854-s001.zip › foods-3461733-supplementary.pdf]

Establishing Detection Methods for Okadaic Acid Aptamer-Target Interactions: Insights from Computational and Experimental Approaches

Wenchong Shan[1, 2, 3], Jiefang Sun[4], Runqing Liu[4], Jing Wang[1, 2, 3,*], Bing Shao[4, 5, 6,*]

1 National Nanfan Research Institute (Sanya), Chinese Academy of Agricultural Sciences, Sanya 572024, China; shanwenchong@163.com
2 Key Laboratory of Agro-Product Quality and Safety, Institute of Quality Standard and Testing Technology for Agro-Products, Chinese Academy of Agricultural Sciences, Beijing 100081, China
3 Key Laboratory of Agri-food Safety and Quality, Ministry of Agriculture of China, Beijing 100081, China
4 Beijing Key Laboratory of Diagnostic and Traceability Technologies for Food Poisoning, Beijing Center for Disease Prevention and Control, Beijing 100013, China; sunjf2001@163.com (J.S.); lrq_srunh@163.com (R.L.)
5 National Key Laboratory of Veterinary Public Health Security, Beijing Key Laboratory of Detection Technology for Animal-Derived Food Safety, and Beijing Laboratory for Food Quality and Safety, College of Veterinary Medicine, China Ag-ricultural University, Beijing 100193, China
6 Food Laboratory of Zhongyuan, Luohe 462300, China

* Correspondence: w_jing2001@126.com (J.W.); shaobingch@sina.com (B.S.); Tel.: +86-010-82106568 (J.W.); +86-010-64407191 (B.S.)

Table S1. Details of FRET-aptamer for marine toxins

Name	Sequence（from 5′ to 3′)	Bases number	Modification	Binding buffer
FRET-OA-1	GGTCACCAACAACAGGGAGCGCTACGCGAAGGGTCAATGTGACGTCATGCGGATGTGTGG	60	5'-FAM+3'-BHQ	BB-01
FRET-OA-2	ATTTGACCATGTCGAGGGAGACGCGCAGTCGCTACCACCT	40	5'-FAM+3'-BHQ	BB-02
FRET-STX-1	GGTATTGAGGGTCGCATCCCGTGGAAACATGTTCATTGGGCGCACTCCGCTTTCTGTAGAT GGCTCTAACTCTCCTCT	78	5'-FAM+3'-BHQ	BB-03
FRET-STX-2	ATAGGAGTCACGACGACCAGCTTTTTACAAAATTCTCTTTTTACCTATATTATGAACAGATA TGTGCGTCTACCTCTTGA	80	5'-FAM+3'-BHQ	BB-02
FRET-STX-3	TTGAGGGTCGCATCCCGTGGAAACAGGTTCATTG	34	5'-FAM+3'-BHQ	BB-04
FRET-STX-4	CATCTGCAGTGTGGCACCATGGAATACGACATTGTTGGCTGTTCTTGGTTATGCGGTTTTTC GTGCTGAGCGTGAATTCGC	81	5'-FAM+3'-BHQ	BB-05
FRET-STX-5	TAGGGAAGAGAAGGACATATGATGGCACAAGGCCTCATCAATCGGTATACGGGTTGACTA GTACATGACCACTTGA	76	5'-FAM+3'-BHQ	BB-06
FRET-TTX-1	ATAGGAGTCACGACGACCAGTCAAATTTTCGTCTACTCAATCTTTCTGTCTTATCTATGTGC GTC TACCTCTTGA	75	5'-FAM+3'-BHQ	BB-02
FRET-TTX-2	GGGAGCTCAGAATAAACGCTCAACCCTGCCGGGGGCTTCTCCTTGCTGCTCTGCTCTGTTC GACATGAGGCCCGGATC	78	5'-FAM+3'-BHQ	BB-07

FRET-TTX-3	AAAAATTTCACACGGGTGCCTCGGCTGTCC	30	5'-FAM+3'-BHQ	BB-08
FRET-MC-1-LR	ATACCAGCTTATTCAATTGGCGCCAAACAGGACCACCATGACAATTACCCATACCACCTCA TTATGCCCCATCTCCGCAGATAGTAAGTGCAATCT	96	5'-FAM+3'-BHQ	BB-02
FRET-MC-2-LA	ATACCAGCTTATTCAATTCACGCACAGAAGACACCTACAGGGCCAGATCACAATCGGTTAG TGAACTCGTACGGCGCGAGATAGTAAGTGCAATCT	96	5'-FAM+3'-BHQ	BB-02
FRET-MC-3-YR	ATACCAGCTTATTCAATTGGACAACATAGGAAAAAGGCTCTGCTACCGGATCCCTGTTGTA TGGGCATATCTGTTGATAGATAGTAAGTGCAATCT	96	5'-FAM+3'-BHQ	BB-02
FRET-MC-4-LR	TTTTTGGGTCCCGGGGTAGGGATGGGAGGTATGGAGGGGTCCTTGTTTCCCTCTTG	56	5'-FAM+3'-BHQ	BB-09
FRET-MC-5-RR	CAGCTCAGAAGCTTGATCCTACTGCCCTTCAATGTTCACTCCTGTTTCCTGATCTTTGTCGA CTCGAAGTCGTGCATCTG	80	5'-FAM+3'-BHQ	BB-01
FRET-BTX-1	GGCCACCAAACCACACCGTCGCAACCGCGAGAACCGAAGTAGTGATCATGTCCCTGCGTG	60	5'-FAM+3'-BHQ	BB-10
FRET-BTX-2	GAGGCAGCACTTCACACGATCTGTGAAGTTTTTGTCATGGTTTGGGGGTGGTAGGGGTGTT GTCTGCGTAATGACTGTAGTGATG	85	5'-FAM+3'-BHQ	BB-11
FRET-GTX-1	AACCTTTGGTCGGGCAAGGTAGGTT	25	5'-FAM+3'-BHQ	BB-12
FRET-DA-1	ATAGGAGTCACGACGACCAGAAAAATAATTTAAATTTTCTACCCAATGCTTTTCGCATAAT ATGTGCGTCTACCTCTTGA	80	5'-FAM+3'-BHQ	BB-02
MST-OA-1	GGTCACCAACAACAGGGAGCGCTACGCGAAGGGTCAATGTGACGTCATGCGGATGTGTGG	60	5'-Cy5	BB-01
MST-OA-2	ATTTGACCATGTCGAGGGAGACGCGCAGTCGCTACCACCT	40	5'-Cy5	BB-02

MST-STX-1	GGTATTGAGGGTCGCATCCCGTGGAAACATGTTCATTGGGCGCACTCCGCTTTCTGTAGAT GGCTCTAACTCTCCTCT	78	5'-Cy5	BB-03
MST-STX-2	ATAGGAGTCACGACGACCAGCTTTTTACAAAATTCTCTTTTTACCTATATTATGAACAGATA TGTGCGTCTACCTCTTGA	80	5'-Cy5	BB-02
MST-STX-3	TTGAGGGTCGCATCCCGTGGAAACAGGTTCATTG	34	5'-Cy5	BB-04
MST-STX-4	CATCTGCAGTGTGGCACCATGGAATACGACATTGTTGGCTGTTCTTGGTTATGCGGTTTTTC GTGCTGAGCGTGAATTCGC	81	5'-Cy5	BB-05
MST-STX-5	TAGGGAAGAGAAGGACATATGATGGCACAAGGCCTCATCAATCGGTATACGGGTTGACTA GTACATGACCACTTGA	76	5'-Cy5	BB-06
MST-TTX-1	ATAGGAGTCACGACGACCAGTCAAATTTTCGTCTACTCAATCTTTCTGTCTTATCTATGTGC GTC TACCTCTTGA	75	5'-Cy5	BB-02
MST-TTX-2	GGGAGCTCAGAATAAACGCTCAACCCTGCCGGGGGCTTCTCCTTGCTGCTCTGCTCTGTTC GACATGAGGCCCGGATC	78	5'-Cy5	BB-07
MST-TTX-3	AAAAATTTCACACGGGTGCCTCGGCTGTCC	30	5'-Cy5	BB-08
MST-MC-1-LR	ATACCAGCTTATTCAATTGGCGCCAAACAGGACCACCATGACAATTACCCATACCACCTCA TTATGCCCCATCTCCGCAGATAGTAAGTGCAATCT	96	5'-Cy5	BB-02
MST-MC-2-LA	ATACCAGCTTATTCAATTCACGCACAGAAGACACCTACAGGGCCAGATCACAATCGGTTAG TGAACTCGTACGGCGCGAGATAGTAAGTGCAATCT	96	5'-Cy5	BB-02
MST-MC-3-YR	ATACCAGCTTATTCAATTGGACAACATAGGAAAAAGGCTCTGCTACCGGATCCCTGTTGTA TGGGCATATCTGTTGATAGATAGTAAGTGCAATCT	96	5'-Cy5	BB-02

MST-MC-4-LR	TTTTTGGGTCCCGGGGTAGGGATGGGAGGTATGGAGGGGTCCTTGTTTCCCTCTTG	56	5'-Cy5	BB-09
MST-MC-5-RR	CAGCTCAGAAGCTTGATCCTACTGCCCTTCAATGTTCACTCCTGTTTCCTGATCTTTGTCGA CTCGAAGTCGTGCATCTG	80	5'-Cy5	BB-01
MST-BTX-1	GGCCACCAAACCACACCGTCGCAACCGCGAGAACCGAAGTAGTGATCATGTCCCTGCGTG	60	5'-Cy5	BB-10
MST-BTX-2	GAGGCAGCACTTCACACGATCTGTGAAGTTTTTGTCATGGTTTGGGGGTGGTAGGGGTGTT GTCTGCGTAATGACTGTAGTGATG	85	5'-Cy5	BB-11
MST-GTX-1	AACCTTTGGTCGGGCAAGGTAGGTT	25	5'-Cy5	BB-12
MST-DA-1	ATAGGAGTCACGACGACCAGAAAAATAATTTAAATTTTCTACCCAATGCTTTTCGCATAAT ATGTGCGTCTACCTCTTGA	80	5'-Cy5	BB-02
OA-2	ATTTGACCATGTCGAGGGAGACGCGCAGTCGCTACCACCT	40	-	BB-02

Table S2. Details of binding buffers used in this study

Name	Salt components	pH
BB-01	50 mM Tris, 150 mM NaCl, and 2 mM $MgCl_2$	7.5
BB-02	50 mM Tris, 150 mM NaCl, and 2 mM $MgCl_2$	7.4
BB-03	10 mM HEPES and 150 mM NaCl	-
BB-04	10 mM PB, 140 mM NaCl, 2.7 mM KCl, and 0.05% Tween-20	7.4
BB-05	20 mM PB, 120 mM KCl, 1 mM $CaCl_2$, and 1 mM $MgCl_2$	7.35
BB-06	20 mM citrate, 100 mM NaCl, 10 mM KCl, and 1 mM $MgCl_2$	3.0
BB-07	10 mM PBS	7.0
BB-08	1 mM PBS	7.4
BB-09	50 mM PBS	7.4
BB-10	50 mM Tris and 10 mM $MgCl_2$	7.5
BB-11	20 mM Hepes, 120 mM NaCl, 5 mM KCl, 1 mM $CaCl_2$, and 1 mM $MgCl_2$	-
BB-12	20 mM Tris-HCl and 10 mM $MgCl_2$	7.5

Table S3. Details of other involved sequences

Name	Sequence（from 5′ to 3′)	Bases number	Modification
APT-mid-BHQ1	ATTTGACCATGTCGAGGGAGACGCGCAG/iBHQ1dT/CGCTACCACCT	40	Mid-BHQ1
FAM-15nt-A	TCGACATGGTCAAAT	15	5'-FAM
FAM-15nt-B	CTCCCTCGACATGGT	15	5'-FAM
FAM-15nt-C	CGCGTCTCCCTCGAC	15	5'-FAM
FAM-15nt-D	GACTGCGCGTCTCCC	15	5'-FAM
FAM-15nt-E	GTAGCGACTGCGCGT	15	5'-FAM
FAM-15nt-F	AGGTGGTAGCGACTG	15	5’-FAM
FAM-15nt-F	AGGTGGTAGCGACTG	15	5’-FAM
FAM-11nt-A	CATGGTCAAAT	11	5'-FAM
FAM-11nt-B	CTCGACATGGT	11	5'-FAM
FAM-11nt-C	TCTCCCTCGAC	11	5'-FAM
FAM-11nt-D	GCGCGTCTCCC	11	5'-FAM
FAM-11nt-E	CGACTGCGCGT	11	5'-FAM

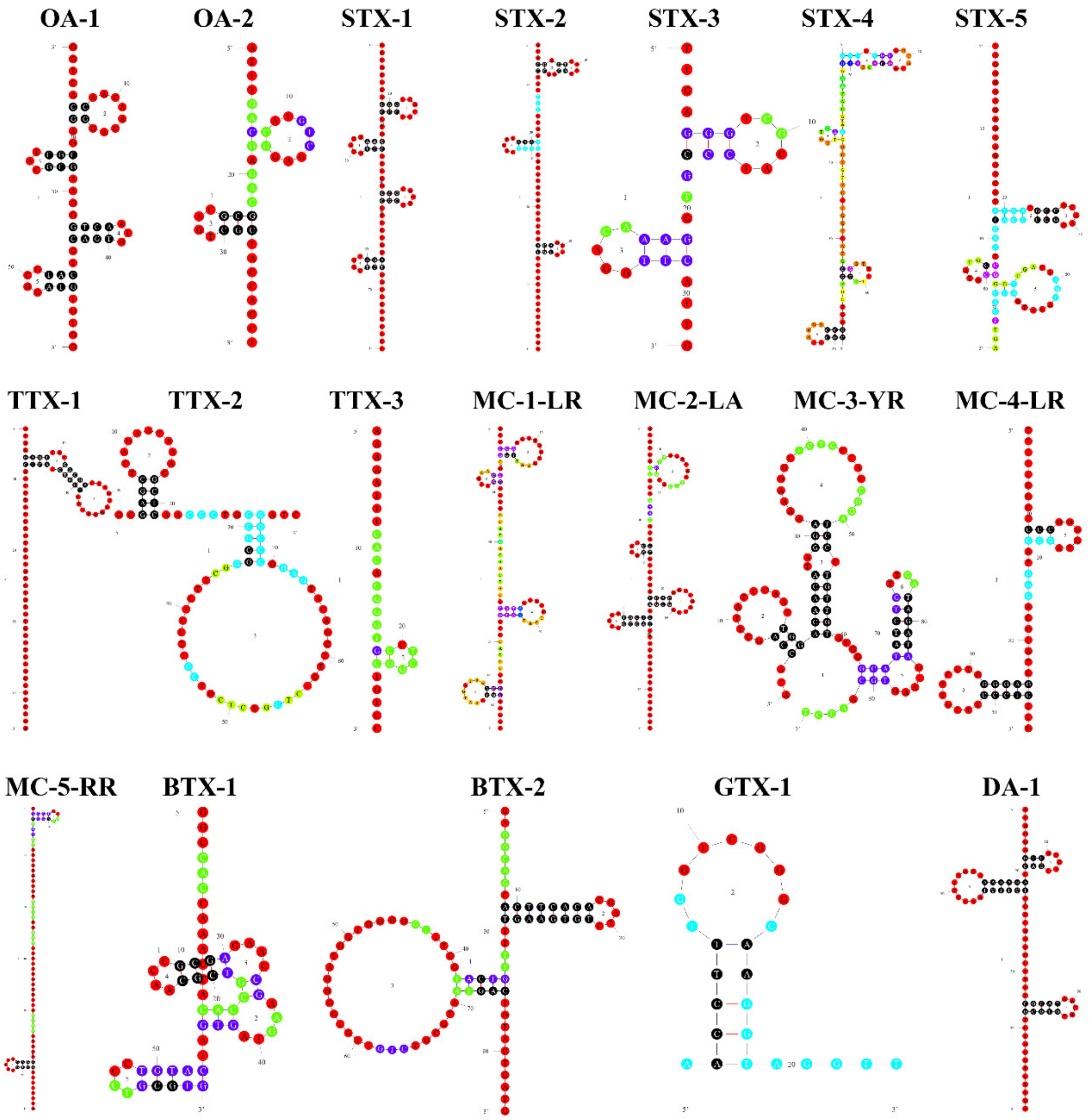

Figure S1. Secondary structures of the marine toxin aptamers with UNAfold prediction with color auto-labeled. Every sequence was input with individual ionic conditions.

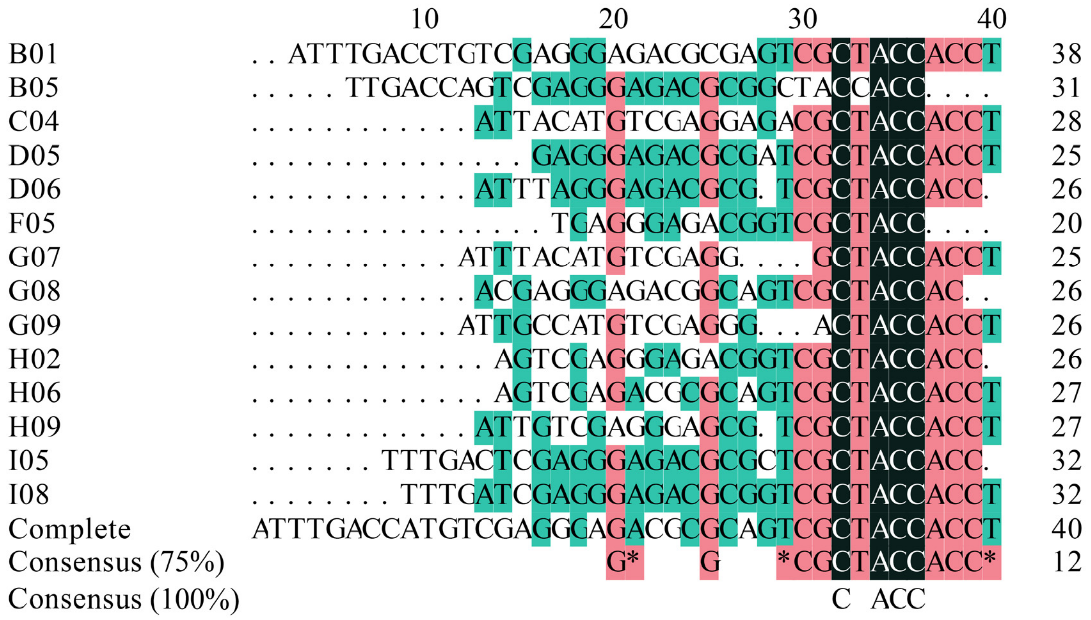

Figure S2. Homology analysis of high score sequences for SYBYL-X core regions prediction used DNAMAN.

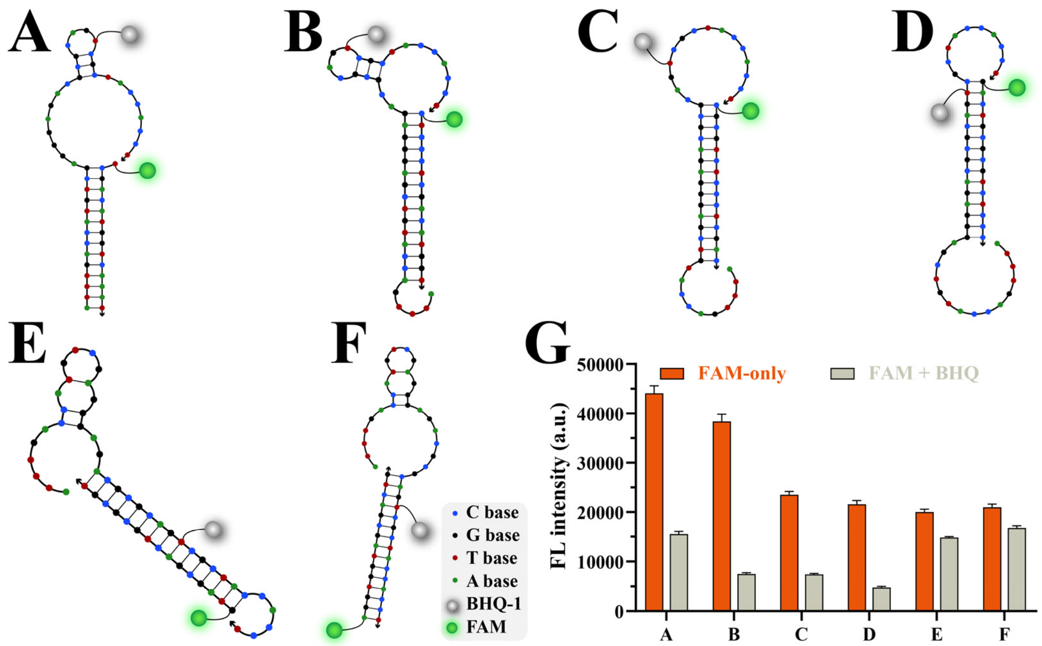

Figure S3. Predicted secondary structures and fluorescence intensity changes in FRET complexes. (A ~ F) The secondary structure predicted by NUPACK (http://www.nupack.org) of DNA strands consisting of the APT-mid-BHQ1 and FAM-15nt-*n*. (G) FI changes of FRET-complexes before and after annealing.

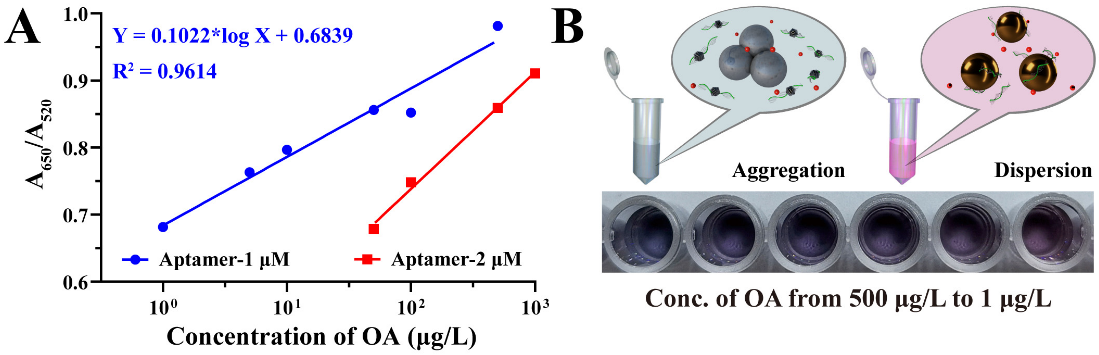

Figure S4. Performance and visual response of aptasensors with varying aptamer and OA concentrations. (A) Performance comparison of aptasensors with different aptamer concentrations. (B) Color change of AuNPs solution with various OA concentrations.

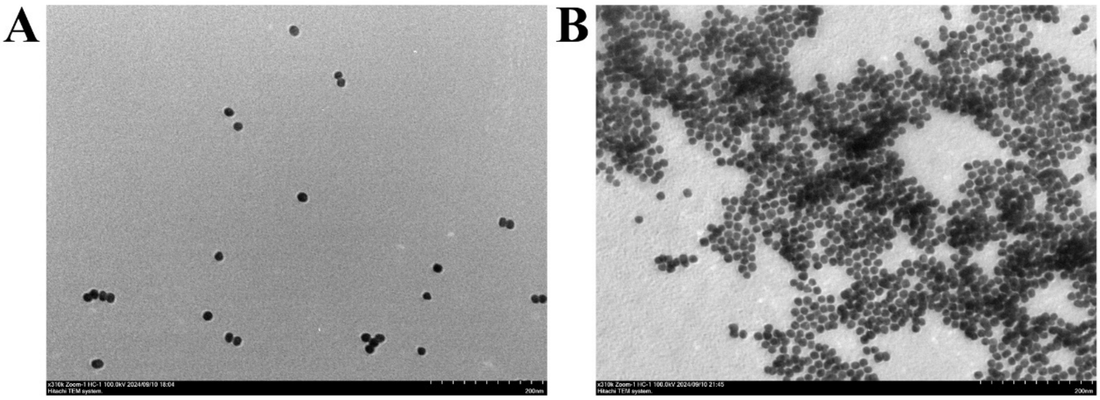

Figure S5. TEM images of AuNP-aptamer system. (A) without OA; (B) with OA addtion.

File S1 (Details of Python code for batch download):

```
import requests
from requests.exceptions import RequestException
import time
import os


def get_one_page(url):
  try:
    response = requests.get(url)
    if response.status_code == 200:
      return response.content
    return None
  except RequestException:
    return None


def get_url(i):
  ###
  left_str = "OA-2"
  ###
  right_str = ".pdb.gz"

  if i <= 9:
    return left_str + "000" + str(i) + right_str
  elif i <= 99:
    return left_str + "00" + str(i) + right_str
  elif i <= 999:
    return left_str + "0" + str(i) + right_str
  else:
    return left_str + str(i) + right_str


def download(url, download_url, dir):
  ###
  path = "./OA-2/" + dir
  ###

  content = get_one_page(download_url)
  if content is None:
    return 0
```

```
  else:
    is_exist = os.path.exists(path)
    if not is_exist:
      os.makedirs(path)
      print(path + 'Successfully Created!')

    ###
    with open("./OA-2/" + dir + "/" + url, 'wb') as f:
      ###
      f.write(content)
      print(url + " download finish")
    return 1


if __name__ == '__main__':
  start_time = time.time()
  ###
  dir_url = ["okA1RLCZhk", "FugGUYR10h", "pdNV91EYsn", "6UyRjgP9W2",
"6FdyUoniyv", "OZdd1AMOfz", "rfwp9neNi6",
       "CeIJJAoeJQ", "SpqQE6f70N", "Xj5z2mImpl"]
  ###
  base_url = "https://www.major.iric.ca/MC-Sym/Work/"
  for dir in dir_url:
    new_url = base_url + dir + "/"
    for i in range(1, 101):
      time.sleep(0.2)
      url = get_url(i)
      download_url = new_url + url
      res = download(url, download_url, dir)
      if res == 0:
        break
  end_time = time.time()
  print("total time: %.2f min" % ((end_time - start_time) / 60))
```
